# Behavior of Weathering Steel in Artificial Harsh Environment

**DOI:** 10.3390/ma17235919

**Published:** 2024-12-03

**Authors:** Tomasz Wierzbicki, Gabriela Rutkowska, Mariusz Żółtowski, Mykola Nagirniak

**Affiliations:** Institute of Civil Engineering, Warsaw University of Life Sciences, 166 Nowoursynowska Street, 02-787 Warsaw, Poland; gabriela_rutkowska@sggw.edu.pl (G.R.); mariusz_zoltowski@sggw.edu.pl (M.Ż.); mykola_nagirniak@sggw.edu.pl (M.N.)

**Keywords:** uncoated weathering steel, steel aging, weld joints, steel strength, impact strength, bridges

## Abstract

The safety and durability of engineering structures, like bridges, which are designed from weathering steels, are conditioned by the development of a sufficiently protective layer of corrosion products. Air pollution, the microclimate around the bridge, the time of wetness, the structural solution of the bridge, and the position and orientation of the surface within the bridge structure all influence the development of protective layers on the surface of the weathering steel. The condition of the formed patina relies on the working conditions of the structure. In fact, it is exposed to various types of salts that appear during the operation of the facility. In this article, the strength parameters of uncoated weathering steel were tested after accelerated aging of welded steel samples in a salt spray chamber. The tests showed the expected degradation of steel after long-term exposure to salt and changes in the strength parameters such as tensile strength, yield strength, and, importantly, impact strength, both in the steel itself and in the elements of the welded connection. The obtained results showed that the change is influenced by both the conditions in which the samples are made (welding method) and the direction of the welded joint (along or across the rolling direction).

## 1. Introduction

Uncoated weathering steel is increasingly used as a construction material in more and more responsible structures [1,2,3]. Due to the specificity of its behavior in corrosive conditions, what is important is not only the strength parameters of this steel achieved at the time of its installation, but above all, the change in these parameters after a certain period of use in difficult conditions. Particularly important are the threats arising from the presence of salt, which disturbs the patina formation process and promotes its destruction [2,4,5,6,7,8,9,10,11]. Bridge structures operate in such conditions and in winter, for road safety reasons, they must be cleaned with de-icing agents [3,12]. Uncontrolled corrosion obviously contributes to the weakening of the engineering value of the entire structure and its individual elements [13,14,15]. It is important to provide appropriate conditions for the formation of a patina and to provide it with adequate protection against damage [16,17,18]. While the corrosion protection techniques are widely used in the petrochemical and gas industries, their implementation in infrastructure structures made of uncoated atmospheric steel remains limited due to the specific environments in which these structures operate and their exposure to atmospheric corrosion [19].

It is obvious that in the case of engineering structures, it is not possible to provide the ideal conditions for the formation of a patina and it is necessary to assess the effects of unfavorable conditions on the behavior of steel. There are several works in the literature on the fatigue strength of aged steels [13,14,15,20] but few studies [21] deal with such a key parameter as impact strength. This parameter is extremely important for structures subject to dynamic loads and operating at low temperatures. These two factors are critical in the development of brittle cracks leading to sudden and widespread disasters. For this reason, in this study, samples of welded joints made using two different welding techniques were subjected to the action of a salt agent causing the accelerated aging of weathering steel. Moreover, welded joints were made in two different workshop situations—welded joints were made along the rolling direction of the steel sheet and across this direction. It turns out that both the technique of making the welded joint and the direction of rolling have a certain impact on the behavior of the strength parameters of steel after aging in a salt chamber.

## 2. The Mechanism of the Formation and Structure of Corrosion Products (Patina) on Weathering Steel Surfaces

At the beginning of the literature review, after introducing the topic of corrosion-resistant steels, it is worth adding an overview of the corrosion mechanisms and patina protection. Works such as [16,17] describe in detail the mechanisms of multilayer patina formation, containing iron and phosphorus compounds, which effectively limit the further corrosion of steel. These studies emphasize the importance of atmospheric factors in the formation and stabilization of the protective layer.

**Figure 1:**
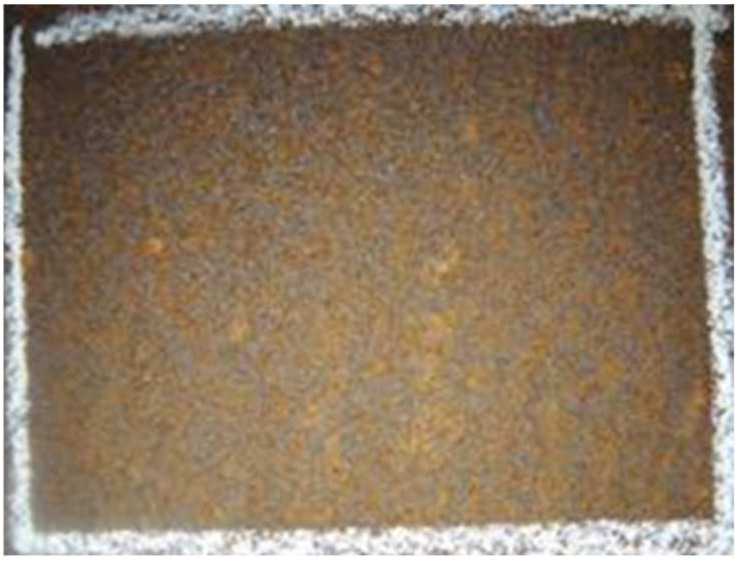
Close view of the weathering steel patina.

Steel with an increased corrosion resistance owes its properties to a thin layer of corrosion products that forms on its surface after the first year of exposure. This is rust whose chemical composition and properties are completely different from the ordinary rust that forms on ordinary carbon steel currently. That is why it is called a patina in the literature. A patina, unlike ordinary rust, whose main component is ferric hydroxide, which is porous and poorly adhered to the steel surface, in addition to the hydroxide, also contains phosphorus sulfur compounds with elements such as copper, chromium, nickel, and molybdenum, depending on the alloying elements added to the hard-to-rust steel, which fundamentally change its character. The microscopical view of patina si given at Figure 1. Over time, the layer of corrosion products becomes tight and poorly permeable to oxygen and other atmospheric reagents, a strongly adhesive coating which, thanks to its properties, inhibits further corrosion processes [22,23,24,25].

It is well known that corrosion is the result of an electrochemical reaction during which metal ions dissolve during cycles of moisture and dryness and form iron hydroxide Fe(OH)x, which precipitates from the solution and creates corrosion products. According to Japanese research [16], a patina consists of two layers. The first one is an external one, composed of FeOOH iron hydroxide with a crystalline structure, and the second one is composed of amorphous (amorphous) iron compounds with hydrogen and oxygen, which adheres directly to the steel surface. The mechanism of formation of the layer adhering to the steel surface has not yet been thoroughly investigated. One of the papers [17] describes the analysis of the rust forming this inner layer being carried out using computer-aided X-ray microanalysis (CMA) and laser spectroscopy on the patina from a sample subjected to a 19-year exposure period. These analyses showed that between the two layers of corrosion products, there is an additional thin intermediate layer consisting of phosphorus compounds. The presence of this film proves that phosphorus, which is an alloy admixture of hard-to-rust steel, changes into phosphatic compounds during patina formation. Observations made during the formation of the colloidal form of the patina using microscopic image recording showed that this layer was formed in the initial period of patina formation. It is assumed that the internal amorphous layer of iron compounds grows into the layer of phosphatic compounds, while the crystalline layer of iron hydroxide (FeOOH) grows above the layer of phosphatic compounds. Research [17] has shown that the internal layer of corrosion products, which provides protection against excessive atmospheric corrosion, consists of amorphous iron oxides containing active alloying ad-mixtures such as chromium, copper, or potassium.

It should be emphasized that in the initial period (up to approx. 6–12 months de-pending on the climatic conditions and the degree of aggressiveness of the environment), there are no significant differences in the process or in the corrosion products occurring on the surface of hard-to-rust steel and low-carbon steel. Later, two layers can be distinguished in the rust patina that forms on hard-to-rust steels: external, similar to the rust of carbon steel and internal, located on the border of the metal–rust phase separation with different compositions and properties. The main component of this layer is hydrated iron oxide (α-FeOOH), composed of small hexagonal or spherical grains, or amorphous, formed from the α-FeOOH variety or directly as a product of iron oxidation, and its formation is influenced by the catalytic influence of the ions present in the layer: Cu^2+^, Cr^3+^_,_ and ${PO}_{4}^{3-}$ [18]. These chemical compounds create a tight, durable layer with good adhesion to the metallic substrate, and therefore provide effective and long-lasting protection against the aggressive effects of the atmosphere. The chemical composition of steel containing alloy additives is a necessary, but not sufficient, condition for the formation of a durable and protective patina. The most important conditions that must also be met include the cyclical nature of the humidification and drying periods and the lack of factors that destroy the already formed patina. The patina layer will not develop if the surface of the sheet is constantly too dry or humid. This is the case, for example, in box girders, where there is moisture due to the lack of air movement. In such a case, even after several years, sheets with traces of uneven corrosion are visible, which locally cause significant corrosion losses. A similar mechanism has the adverse effect of sodium chloride on the formation and durability of the patina. It turns out that the presence of salt on the surface of the sheet reduces the wetting of the steel surface. Salt crystals hygroscopically bind moisture from the air, lowering the relative humidity to approximately 55%, at which extremely aggressive corrosion is accelerated [1]. Moreover, at high concentrations or in the presence of other corrosive factors, salts penetrate the patina layer, reaching the metal–patina boundary, where they create new corrosion products, causing the normal patina to detach. As a result of this phenomenon, the delamination of flakes or entire patina sheets is observed [20]. Salts are not the only threat to the durability of the formed patina and the loss of its protective properties. This includes rinsing with water, which is considered the best solvent (the famous drop that hollows out the rock), the contamination of the steel surface with chlorides that dissolve the patina, and the presence of impurities that constantly retain moisture (mud, coal dust, bird nests, and droppings). Properly produced and protected against de-grading factors, a patina, after five years of exposure, is able to stabilize the corrosion losses at a level of several to a dozen μm per year, depending on the aggressiveness of the environment [26].

The literature review can be concluded with a discussion of the works on the modern methods of the monitoring and intelligent detection of the wear of corrosion-resistant steels, which can be an interesting addition and a guide for further research. For example, Ji et al. [23] conducted a comprehensive review of the monitoring methods, damage detection, and intelligent detection systems.

The review of previous studies reveals a knowledge gap regarding the combined effect of the welding methods and weld orientation on the degradation of corrosion-resistant steels in extreme corrosive conditions. Despite the numerous studies on the chemical composition of patinas and their stability in various environments, studies rarely consider the mechanical factors resulting from the welding process, as well as the effect of the weld orientation on maintaining the integrity of the protective layer. This study fills this gap by combining the analysis of the microstructural and mechanical effects with the assessment of the corrosion resistance of welded joints in a saline environment.

Therefore, this study addresses the following research questions:

- What is the significance of the choice of welding method (arc welding with a consumable electrode and active gas welding) for the mechanical strength and corrosion resistance of weathering steels under artificial aging conditions?
- How does the orientation of the weld with respect to the rolling direction affect the formation of a protective patina and its stability under accelerated aging?
- How does the combination of the welding method and weld orientation affect the microstructural changes and long-term performance of weathering steels in salt environments?

## 3. Structural Weathering Steel-Welded Joints

Structural steel is used in many engineering applications like bridges, transmission power masts, building frame structures, or tankers and siloes. The primary method used for joining steel sheets is welding. It is well known that the welding process negatively influences the steel properties, and special attention must be paid to the welding techniques applied for fixing including the welding materials and welding process parameters (time of welding, temperature control as well as straightening techniques applied after welding). Both the literature review and the research studied revealed a certain information gap regarding the testing of welded joints made of rust-resistant steels that have undergone severe corrosion. It was noticeable that there were no tests of a joint subjected to corrosion processes that had been operating for a long time in real environmental conditions. The thesis of the research was therefore to state that despite subjecting sheets of hard-to-rust steel to local welding treatment, the modern welding materials and appropriately selected welding parameters allow the condition of durability to be met despite the long-term exposure to unfavorable conditions favoring corrosion.

### 3.1. Material Selection for the Test Samples’ Preparation

The S355J2W+N steel was selected to make the samples of welded joints. The samples were prepared from one sheet of metal with the dimensions of 1600 × 680 × 25 mm made at the Vitkovice Steel a. s., Ostrava, Czech Republic. Based on the certificate, it is possible to present in the Table 1. the chemical composition of the steel from which the sample sheet was made:

The certificate also provides the mechanical properties of the steel used to prepare the samples:

- Minimum yield strength R_eH_ = 397 MPa;
- Minimum immediate strength R_m_ = 555 MPa;
- A_5_ narrowing = 26.3%.

**Figure 2:**
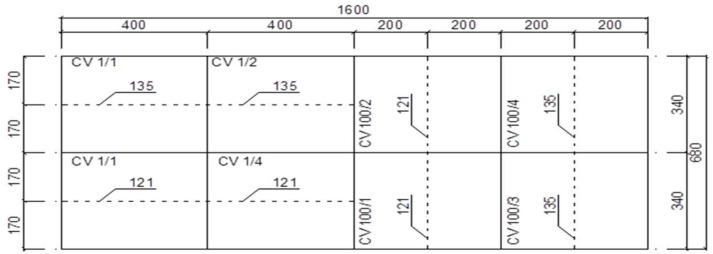
Schematic partition of steel sheet prepared for testing.

A sheet of metal measuring 1600 × 680 mm was cut into sixteen elements, eight of which had the dimensions of 400 × 170 mm, and the remaining eight 340 × 200 mm. This division resulted from the need to prepare samples for welded joints, assuming that half of them would be welded across the direction of rolling, and half of them perpendicularly. Figure 2 shows a schematic division of the sheet for testing along with the designation of the welding methods.

#### Welding Method

One of the two welding technologies adopted in the experiment was submerged arc welding with electrode wire (method 121). In accordance with the Welding Procedure Guidelines for welding S355J2W+N steel sheets with thicknesses from 15 to 60 mm using this method, AUTOROD 13.36. wire was used to connect the sheets.

The certificate provides the mechanical properties of the wire used in the submerged arc welding technology used to prepare sample nos. CV 1/3, CV 1/4, CV 100/1, CV 100/2:

- Minimum yield strength R_eH_ = 549 MPa;
- Minimum immediate strength R_m_ = 636 MPa;
- Minimum narrowing A_5_ = 26.0%;
- Minimum breaking work 82 J.

On the basis of the certificate, it is also possible to provide the chemical composition of the AUTOROD 13.36 wire as presented in Table 2.

The second welding technology adopted in the experiment was the technology of welding with a consumable electrode in the shield of active CO_2_ gases with argon (method 135). In accordance with the Welding Procedure Guidelines, a NICOR electrode was used to weld S355J2W+N steel sheets with a thickness of 15 to 60 mm using this method.

The certificate provides the mechanical properties of the electrode used in the consumable electrode welding technology in the shield of active CO_2_ gases with argon, used to prepare sample nos. CV 1/1, CV 1/2, CV 100/3, CV 100/4:

- Minimum yield strength R_eH_ = 580 MPa;
- Minimum immediate strength R_m_ = 640 MPa;
- Minimum narrowing A_5_ = 26.0%;
- Minimum breaking work 47 J.

The chemical composition of the NICOR electrode based on the certificate is given in Table 3.

## 4. Materials and Methods

### 4.1. Samples’ Preparation

It was decided to prepare four types of samples using the two welding methods. Thus, four samples of welded joints were made using the submerged arc welding technology (method 121) and another four using the MAG welding technology with a consumable electrode in the shield of active CO_2_ gases with argon (method 135), where for half of the samples in each group, the welding bead was arranged perpendicular, and for the other half parallel, to the rolling direction. The welding methods were chosen due to their frequent use in weathering steel bridge structure manufacturing.

The samples were marked according to the following key:

- Samples welded along the rolling direction as follows:
  ○ CV 1/1—welded using method 135;
  ○ CV 1/2—welded using method 135;
  ○ CV 1/3—welded using method 121;
  ○ CV 1/4—welded using method 121.
- Samples welded across the rolling direction as follows:
  ○ CV 100/1—welded using method 121;
  ○ CV 100/2—welded using method 121;
  ○ CV 100/3—welded using method 135;
  ○ CV 100/4—welded using method 135.

**Figure 3:**
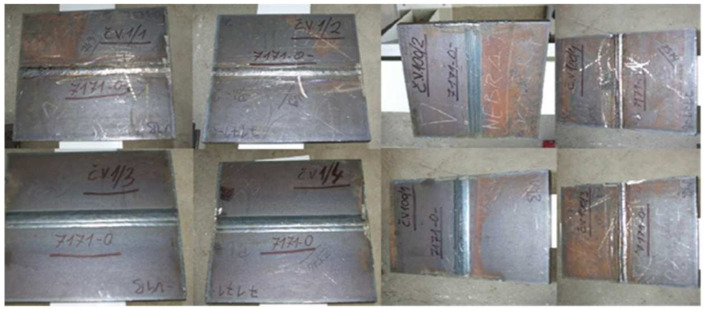
General view of the steel sheet cut and welded for testing samples.

The partition of the sheet and the general view of the samples are shown in Figure 3.

**Figure 4:**
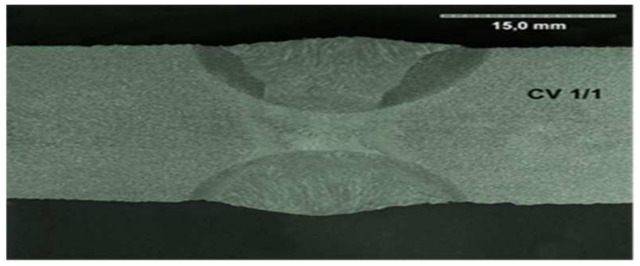
The cross section of welded joint sample CV 1/1.

**Figure 5:**
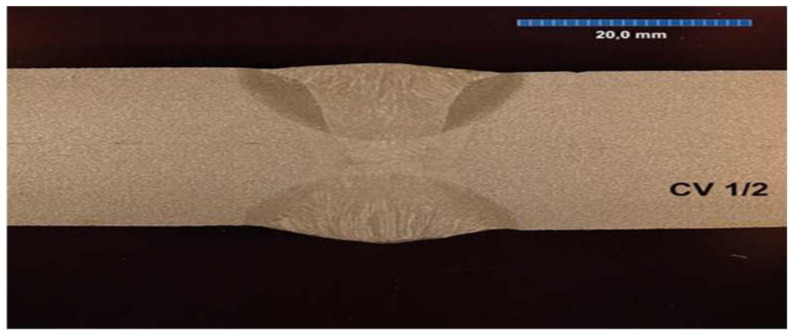
The cross section of welded joint sample CV 1/2.

**Figure 6:**
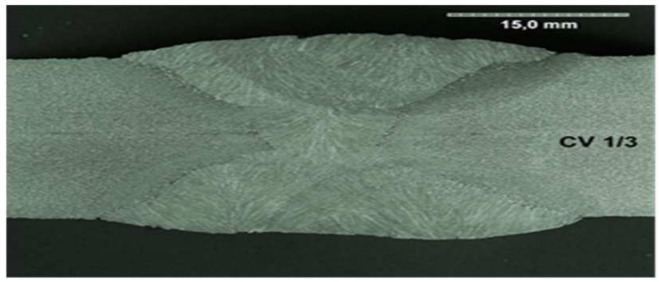
The cross section of welded joint sample CV 1/3.

**Figure 7:**
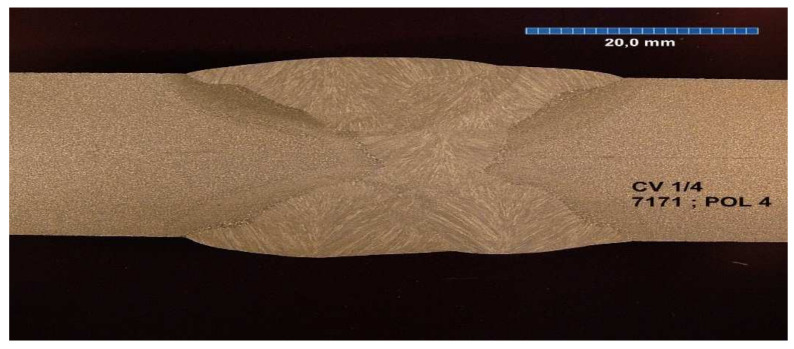
The cross section of welded joint sample CV 1/4.

**Figure 8:**
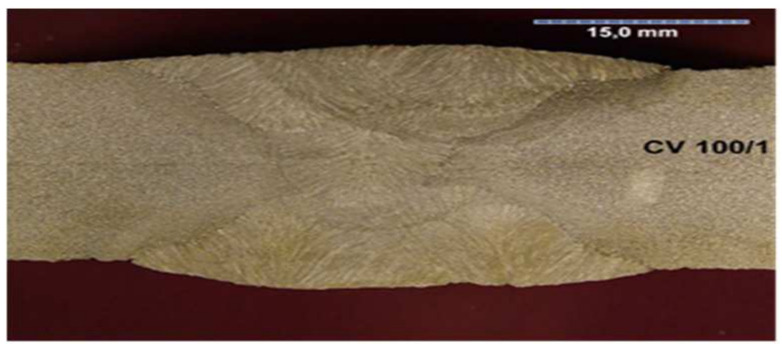
The cross section of welded joint sample CV 100/1.

**Figure 9:**
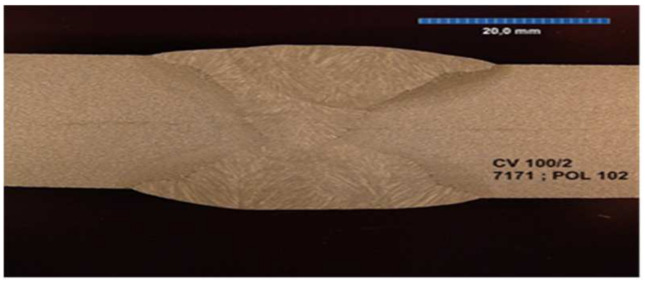
The cross section of welded joint sample CV 100/2.

**Figure 10:**
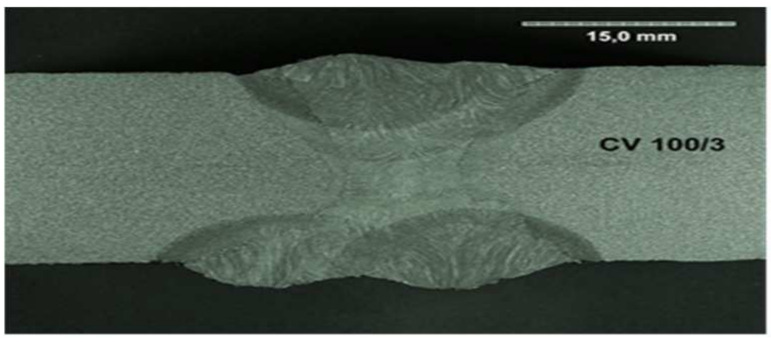
The cross section of welded joint sample CV 100/3.

**Figure 11:**
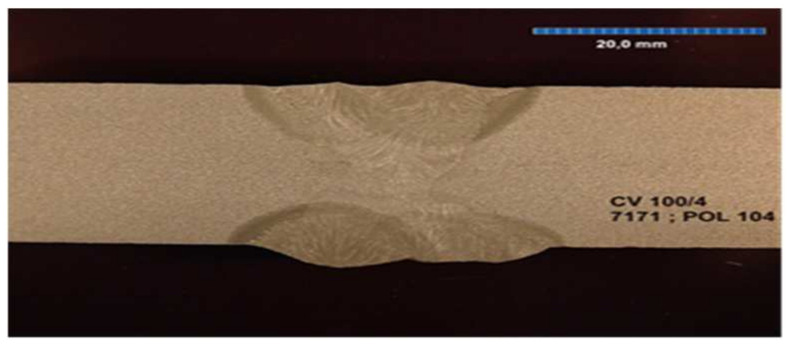
The cross section of welded joint sample CV 100/4.

All the samples were made at the same time, in the same factory, and were stored in the same climatic conditions until the experiment began. All the welded joints were subjected to ultrasonographic and radiographic tests. The welds did not show any defects or damage that could weaken the tested connection. Microscopic photos of the cross-section of individual joints were also taken. These photos are shown in Figure 4, Figure 5, Figure 6, Figure 7, Figure 8, Figure 9, Figure 10 and Figure 11.

**Figure 12:**
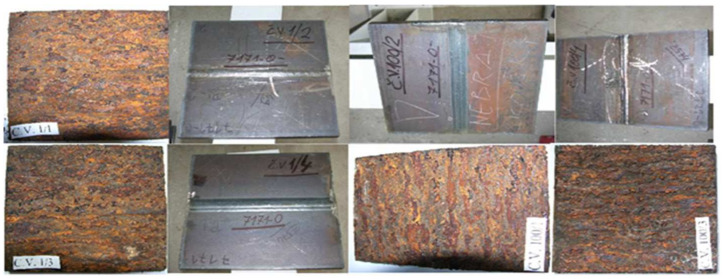
General view of the tested steel sheet sampled as delivered and subjected to artificial maturing.

The samples were divided into two groups (Figure 12). The first one, including the samples marked CV 1/1, CV 1/3, CV 100/1, and CV 100/3, was placed in a salt chamber in which it was bathed in a salt mist created with a 5% NaCl solution in water, at a temperature of 35° C. A BS1-type salt chamber manufactured by Elcometer (Manchester, UK) was used to prepare the samples.

The total time of keeping the samples in the salt chamber was 1440 h, which corresponds to the work in a steel structure for over 15 years in the most severe corrosive atmosphere, C5-M, determined according to [27].

The measurements showed that comparative samples made of the same material and kept in the same conditions lost from 3.6% to 4.7% of their cross section and from 4.6% to 7.5% of their mass. These results are presented in Table 4 and Table 5. These results indicate severe corrosion of the samples and the failure of the mechanism to develop a protective patina, which is a beneficial phenomenon in a given experiment, as it allows the simulation of extremely unfavorable operating conditions of steel elements in real conditions. Despite the clear rules for the use of rust-resistant steels, it often happens that the sheet metal is exposed to direct and continuous exposure to salty water (e.g., in the case of a leaky expansion joint), which not only prevents the proper patina from forming, but also effectively destroys the already formed patina. Therefore, the experiment attempted to model such an unfavorable case.

### 4.2. Strength Tests of Samples as Delivered and Artificially Aged

The purpose of the research was to check the impact of corrosion (due to artificial aging) on the quality and condition of the welded joint connecting sheets of hard-to-rust steel. The experience gained on some objects made of rust-resistant steels leads some of their users to blame these steels for the damage to welded joints, as they undergo uncontrolled corrosion when the patina formation process is disturbed. Moreover, these users, somewhat rightly, believe that the inspection and early detection of defects in such joints in the case of rust-resistant steels is difficult due to their appearance. Demonstrating that this type of connection is not particularly susceptible to damage could be a valuable tip when deciding on the choice of material for bridge structures.

To prove this assumption, a series of tests were carried out, consisting of the measurement of the immediate and bending strength, the measurement of the impact strength in several zones of the welded joint, and the measurement of the hardness in several cross sections of the weld and the base material adjacent to the joint. The tests were carried out for all four types of connections and for both cases of the steel condition—as delivered and after artificial aging (corroded).

The test was carried out on all the samples, both those as delivered and after artificial aging. The testing of the samples as delivered (CV 1/2, CV 1/4, CV 100/2, and CV 100/4) was performed on 18 February 2010, and the testing of the samples subjected to artificial aging (CV 1/1, CV 1/3, CV 100/1, and CV 100/3) was performed on 5 August 2010. All the test results were considered positive and met the requirements of the standard [28].

The tests were performed on a ZD100 testing machine no. 283/22, with a range of 0–400 kN and an elementary division of 2.0 kN. The tests were carried out at a temperature of +20 °C. The tests were carried out each time on two square samples with the dimensions of 25 mm, taken from test sheets connected with a butt weld. The test was carried out by bending the sample through an angle of 180° on a mandrel with a diameter equal to 3 sheet thicknesses (75 mm).

The tests were performed using a Charpy hammer of the IMPACT450 type, registration number V9GD, with a range of 0–450 J with an elementary division of 0.1 J. The tests were carried out at a temperature of −20 °C. The tests were each carried out on three samples with a rectangular cross section of 10.0 × 8.0 mm with a V-notch, taken from test sheets connected with a butt weld. The test was carried out for samples taken in three characteristic places of the welded joint: in the weld, in the heat affected zone, and in the fusion area. Each time, the arithmetic mean was taken from the three results, which was the official result of the test. All the test results were considered positive and met the requirements of the steel standard [28].

Similar results were obtained in [21], where in the studies on the behavior of atmospheric steel 09CuPCrNi, a significant influence of the rolling direction and welding method on the corrosion resistance of welded joints in a simulated marine environment was shown.

## 5. Results

### 5.1. Strength Tests of the Samples as Delivered and Artificially Aged

**Figure 13:**
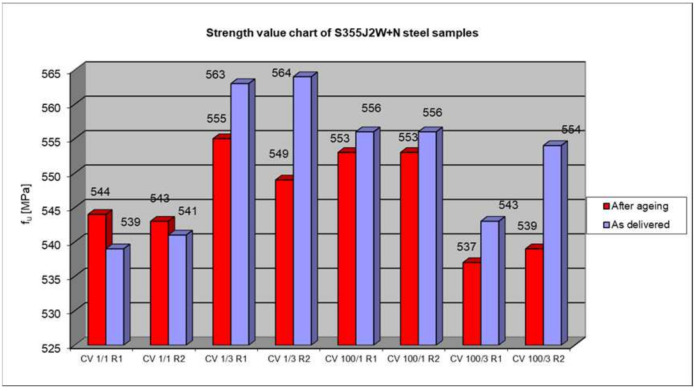
Comparison of summary of strength testing results of a welded joint—as delivered and after accelerated aging.

**Figure 14:**
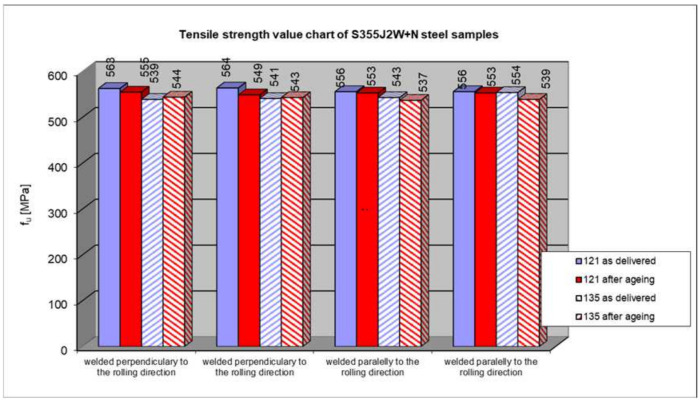
Comparison of summary of strength testing results of a welded joint—rolling direction of samples.

**Figure 15:**
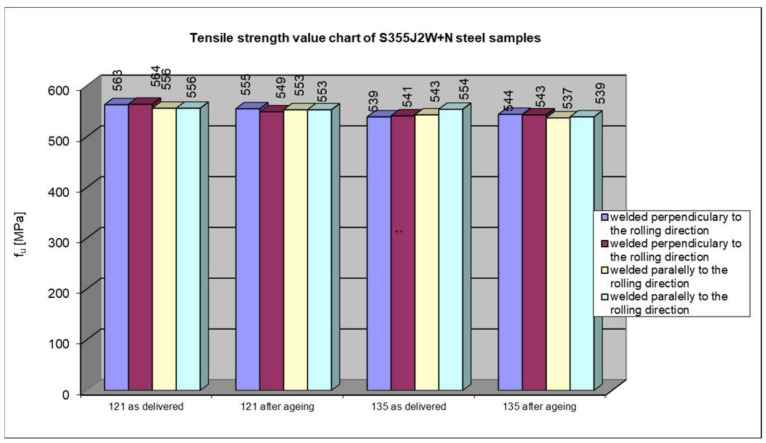
Comparison of the results of strength tests depending on the welding technology and the condition of the S355J2W+N steel sample.

Summary of the strength testing results of a welded joint. The results are presented at Figure 13, Figure 14 and Figure 15.

The course of tensile tests of the welded joint samples was similar in all the cases, i.e., the connection rupture occurred outside the weld, in the native material. Therefore, the test results in terms of the influence of artificial aging on the temporary strength can only be applied to S355J2W+N steel. This also proves that the welding technology had no impact on the load-bearing capacity of the connection. The analysis of the results of the tensile test of the welded joint does not allow for a definite determination of the influence of the rolling direction on the load-bearing capacity of the joint. Also, the third factor taken into account when modelling the experiment, i.e., material aging, did not significantly adversely affect the final result. In six out of eight cases, there was a reduction in the short-term strength (from 0.5% to 2.7%). At the same time, in the other two cases, the steel that did not rust easily after staying in the salt chamber had a higher strength (by 0.4% and 0.9%), and both samples were taken from the same batch of the CV 1/1 and CV 1/2 samples with the axis transverse to the rolling direction). Taking into account that all the tested samples showed a temporary strength higher (from 119.3% to 125.3%) than that required for this type of hard-to-rust steel (470 MPa) and the fact that the differences in results in individual cases are insignificant (max 2.7%), it can be concluded that the fact of severe corrosion caused by artificial aging in a salt spray chamber did not have a significant impact on the temporary strength of the tested welded joint.

### 5.2. The Bending Strength of the Welded Joint Testing Results

A total of twenty-four bending strength tests of the welded joint were performed. All the tests were successful because all the samples were bent on a mandrel with a diameter of three times the thickness of the sheet (75 mm) through an angle of 180°. After the test, no cracks or scratches were observed on the surface of the samples. It should be added that, in accordance with the Polish standard [28], it was required to bend the sample without damage to an angle of only 150°.

Also based on this test, it is possible to conclude that the degree of corrosion of the welded joint has no influence on the bending strength. Due to the positive test results, it was also impossible to determine the influence of the other factors considered in the experiment (welding technology and rolling direction) on the bending strength.

### 5.3. Impact Test Results of the Butt Joint of the Test Plates

Three types of impact tests were carried out on each type of welded joint sample made of rust-resistant steel S355J2W+N: impact tests in the heat-affected zone (HAZ), in the fusion line, and in the weld itself. Three measurements were carried out for each type of test, and in the case of the weld impact test for steel as delivered, three additional measurements were made (a total of six for this case). The test results for each type of study are presented below.

### 5.4. Impact Test Results in the Heat-Affected Zone (Haz)

**Figure 16:**
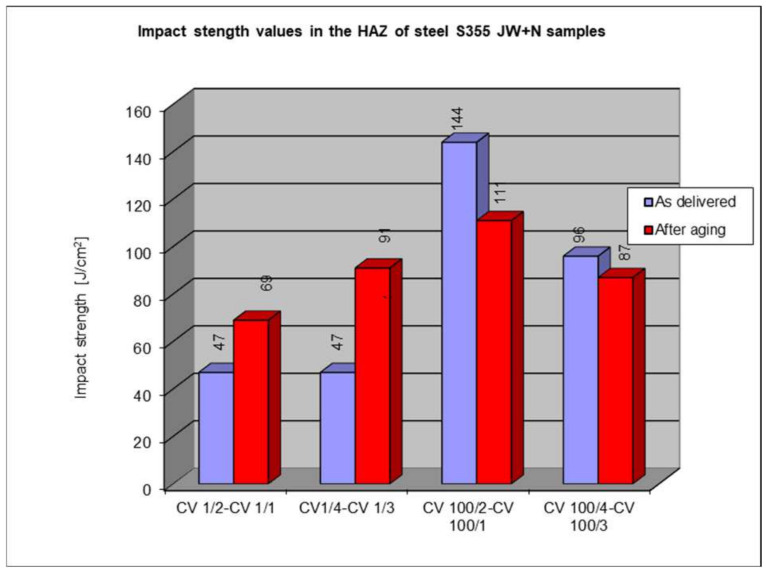
Graphical representation of average impact strength results measured in the heat-affected zone of the HAZ.

**Figure 17:**
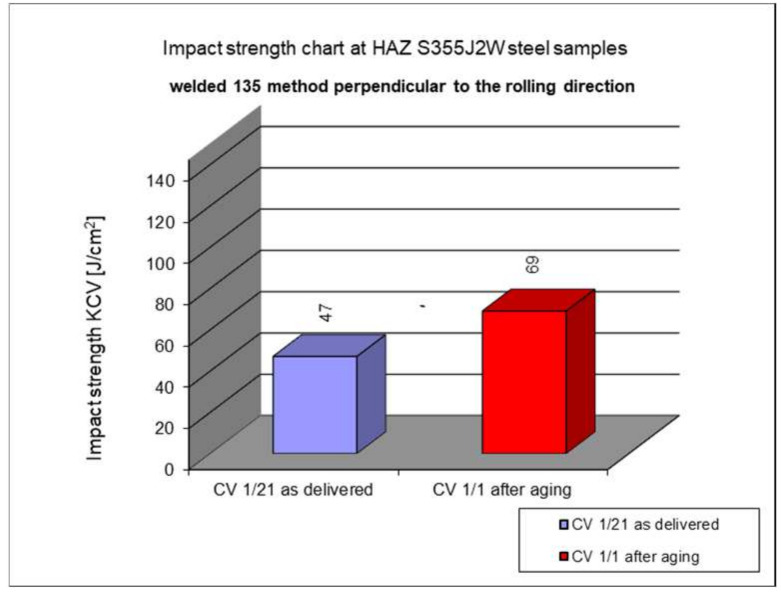
Graphical representation of average impact strength results measured in the heat-affected zone of the HAZ, 135 welding technology (perpendicular to the rolling direction).

**Figure 18:**
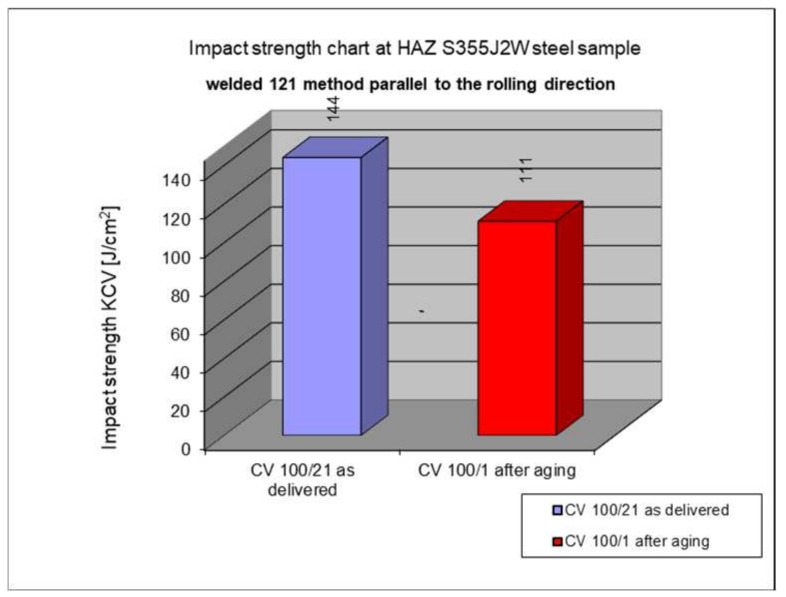
Graphical representation of average impact strength results measured in the heat-affected zone of the HAZ, 121 welding technology (in parallel to the rolling direction).

**Figure 19:**
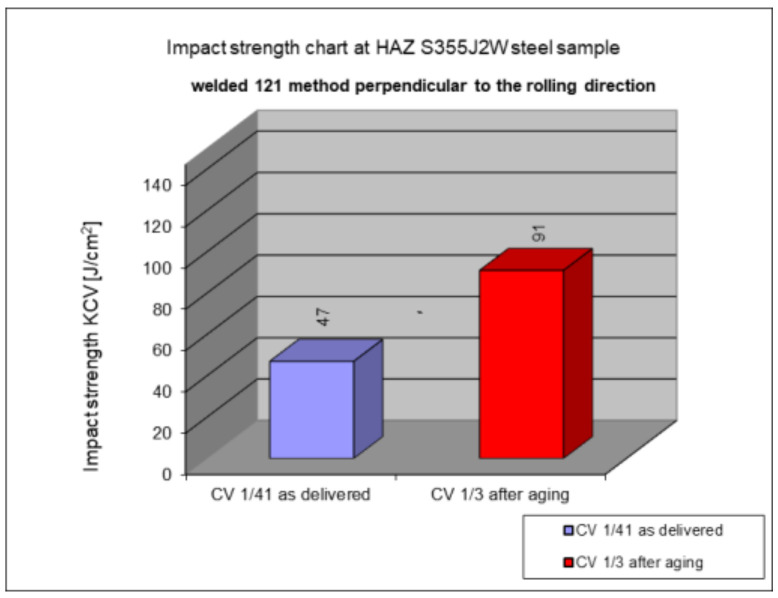
Graphical representation of average impact strength results measured in the heat-affected zone of the HAZ, 121 welding technology (perpendicular to the rolling direction).

**Figure 20:**
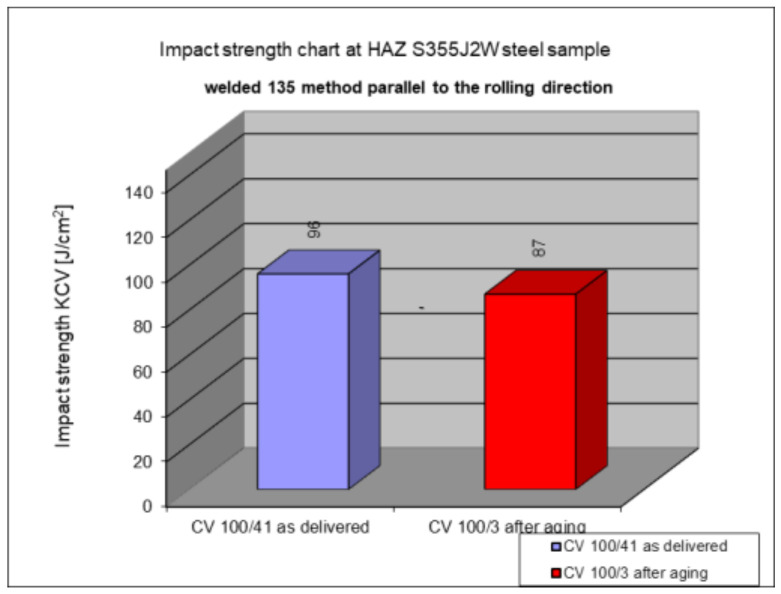
Graphical representation of average impact strength results measured in the heat-affected zone of the HAZ, 135 welding technology (in parallel to the rolling direction).

The following subsection presents the results of impact testing in the heat-affected zone (HAZ) of a welded joint made of S355J2W+N hard-to-rust steel. The results are given in Table 6. and presented at Figure 16, Figure 17, Figure 18, Figure 19 and Figure 20.

### 5.5. Impact Test Results in the Weld

**Figure 21:**
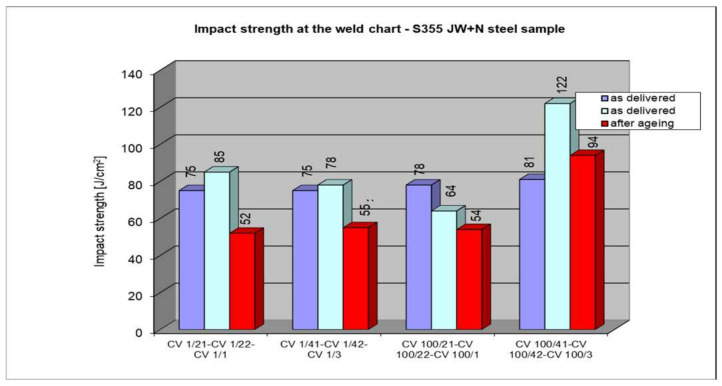
Graphical representation of average impact strength results measured in the weld.

**Figure 22:**
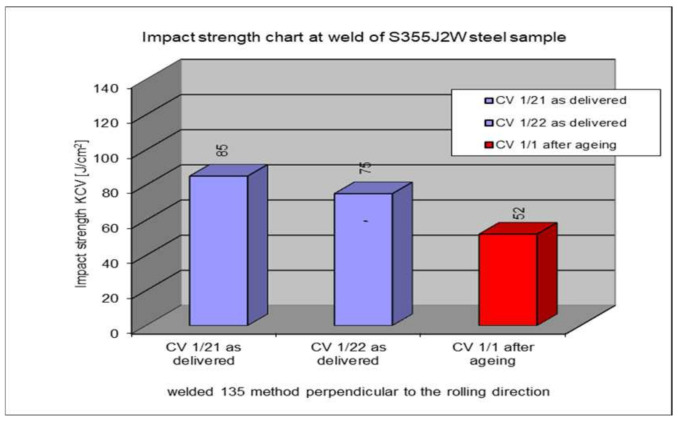
Graphical representation of average impact strength results measured in the weld for 135 welding technology.

**Figure 23:**
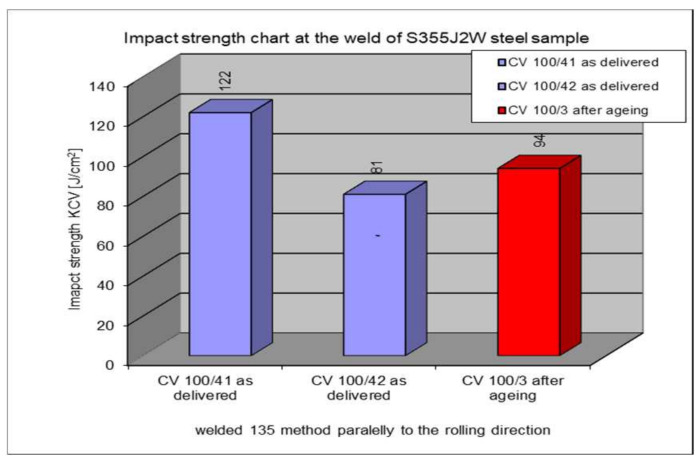
Graphical representation of average impact strength results measured in the weld for 135 welding technology.

**Figure 24:**
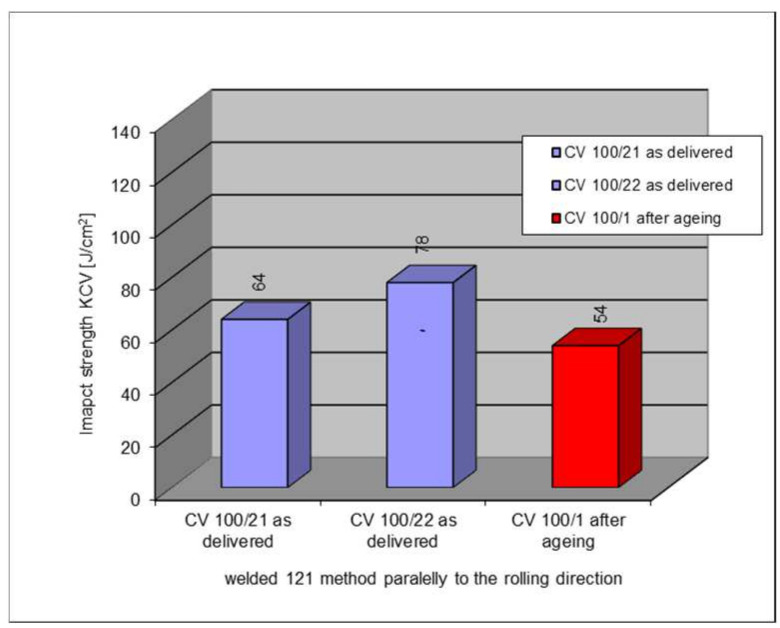
Graphical representation of average impact strength results measured in the weld for 121 welding technology.

**Figure 25:**
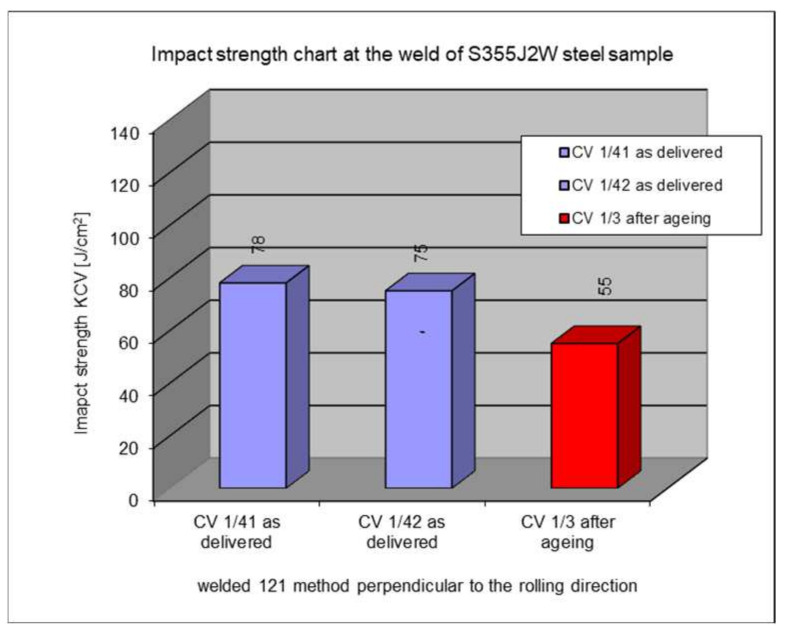
Graphical representation of average impact strength results measured in the weld for 121 welding technology.

The following section presents the results of impact strength testing in the weld of a welded joint made from a S355J2W+N rust-resistant steel sheet. The results are given in Table 7. and presented at Figure 21, Figure 22, Figure 23, Figure 24 and Figure 25.

### 5.6. Impact Test Results—Comparison of the Impact Strength in All the Elements of the Welded Joints

**Figure 26:**
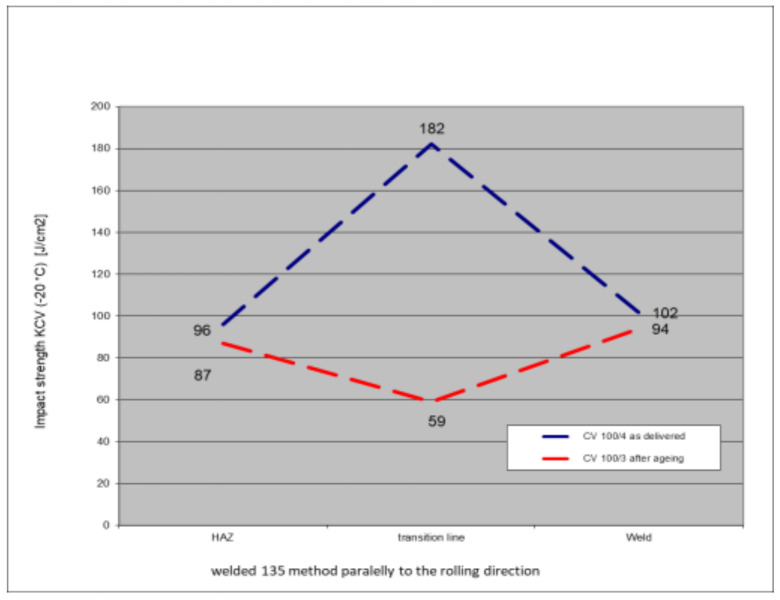
Graphical presentation of the average impact strength results measured in individual elements of the welded joint for welding technology 135 (along the rolling direction).

**Figure 27:**
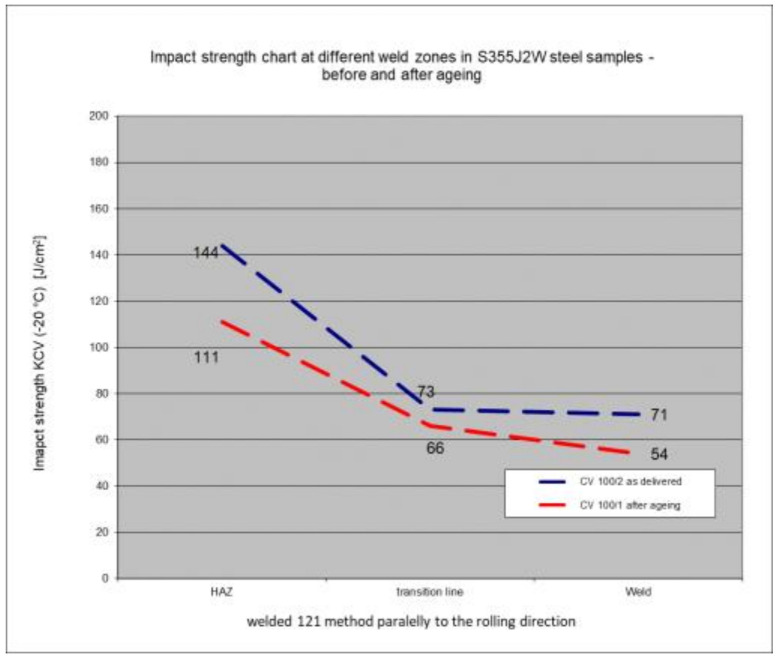
Graphical presentation of the average impact strength results measured in individual elements of the welded joint for welding technology 121 (along the rolling direction).

**Figure 28:**
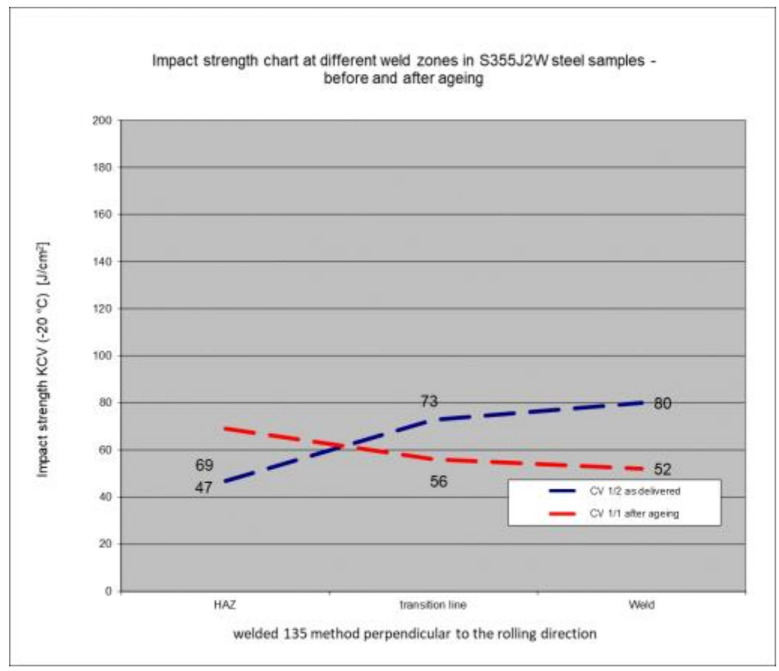
Graphical representation of the average impact strength results measured in individual elements of the welded joint for welding technology 135 (across the rolling direction).

**Figure 29:**
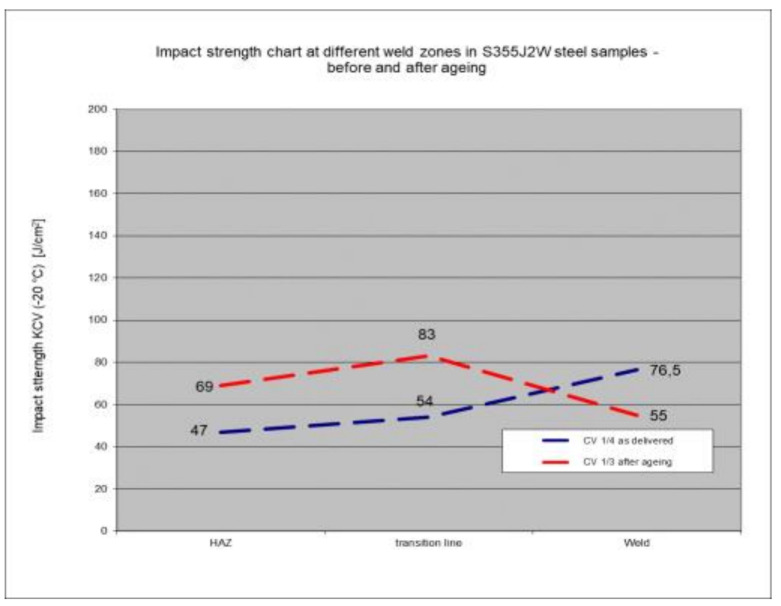
Graphical representation of the average impact strength results measured in individual elements of the welded joint for welding technology 121 (across the rolling direction).

The above subsections present the results of the impact tests of the welded joint elements in a numerical and graphical form. These results were presented separately for each fragment of the connection. The results for all the elements of the welded joint are presented graphically below at Figure 26, Figure 27, Figure 28 and Figure 29, as well as a comparison of the results before and after artificial aging of the S355J2W+N steel sheet.

## 6. Discussion

Based on the analysis of all the results presented in this work, it can be concluded that all the elements of the welded joint in a significant state of corrosion and with the tested joining technologies showed a KCV impact strength higher than that required by the Polish standard [28] for this type of structure. The minimum KCV impact strength at −20 °C should not be lower than 35 J/cm^2^.

Despite the positive test result, it seems that the dependence of the KCV impact strength value on the type of connection, corrosion condition, or the direction of the rolling of the connection cannot be determined in the case of measurements in the heat-affected zone (HAZ) and the fusion line. However, a decrease in the KCV impact strength can be observed in the weld of the artificially aged sample. For the most unfavorable case, it was 38.8%, while the smallest drop in impact strength was 15.6%. At the same time, an incidental increase in the KCV impact strength by 16.0% was noted after artificial aging in the case of sample CV 100/4KV11-31 (a sheet of hard-to-rust steel S355J2W+N welded using the 135 technology, in the direction transverse to the rolling direction).

In summary, the studies have shown that the corrosion of atmospheric steel in a high-salinity environment significantly affects its strength properties. In particular, it was found that the presence of salt and humidity significantly accelerate the degradation of the patina layer, causing a loss of thickness and mass of the samples. An important finding is also the effect of the welding technology and rolling direction on the corrosion resistance of the weld, which indicates the need for the precise selection of the welding parameters for the structures used in aggressive environmental conditions.

As far as engineering practice is concerned, the results of the research on the corrosion of atmospheric steel in a high-salt environment have significant practical significance, in particular as follows:

- The identified factors influencing the accelerated degradation of steel can be taken into account in the design of bridges, building facades, and offshore structures.
- The research results can help engineers make decisions on the selection of the appropriate welding technology and rolling direction, which will increase the durability of the structure.
- The importance of controlling the operating environment, especially in areas exposed to chloride contamination, may lead to the use of additional corrosion protection techniques, such as protective coatings or cathodic corrosion protection.

These results are also useful in the development of the technical standards and recommendations for the construction industry, especially in the field of securing infrastructure in road (winter time) and coastal areas.

## 7. Conclusions

The above presented results prove that weathering steel can be used as a full-fledged construction material for the construction of structures exposed to high salinity environments, both periodically, like bridges suffering from dicing pollution during the winter time, and permanently, like the structures located in coastal areas. The aging of steel in these conditions does not significantly change the mechanical properties of the steel nor welded joints, and the only problem remains the possible loss of cross section due to the destruction of the patina.

The results of the study allow for the formulation of the following key conclusions:

1. The effect of a salt environment on mechanical properties: The exposure of steel to an aggressive salt environment leads to a significant degradation of its mechanical properties, especially in welded joints in the direction transverse to rolling. The weakening of the parameters such as tensile strength and yield strength was observed, which may affect the safety of structures subjected to dynamic loading in difficult environmental conditions. The accelerated aging process simulates corrosive phenomena that may occur in structures exposed to long-term salt exposure in real operating conditions.
2. The role of welding technology in corrosion resistance: The results indicate that the choice of welding technology significantly affects the corrosion resistance of joints. The samples welded via the submerged arc method showed better strength parameters after aging compared to those welded via the MAG method, which suggests that the submerged arc method may be more suitable for structures exposed to aggressive environments. Further research should focus on optimizing the welding parameters for corrosion-resistant steels.
3. The degradation of the patina in the presence of salt: The protective patina layer that normally protects steel from corrosion degrades in a high-salt environment, significantly reducing its protective properties. This phenomenon indicates that even corrosion-resistant steels may require additional protection in situations where they are exposed to intense salt attack (e.g., in marine climates or in areas with intensive road salting).
4. The importance of welding orientation relative to rolling: Studies have shown that the welding direction relative to the rolling direction of the steel has a significant effect on the strength of joints after accelerated aging. The joints welded in the rolling direction showed higher resistance to mechanical degradation compared to the joints welded transversely, suggesting that the welding orientation should be taken into account when designing structures, especially those subjected to dynamic loads.

Further research is recommended to optimize the chemical composition of the patina and to investigate different welding techniques that could improve the corrosion resistance of steel structures in high-salt environments. It is also worthwhile to continue the research on the surface modifications of the patina to increase its resistance to atmospheric agents and to apply cathodic protection techniques as a potential complement to the protective layer.

## Figures and Tables

**Table 1:** Steel sheet chemical composition made of S355J2W+N grade used for samples.

Alloying Elements [%].															
C	Mn	Si	P	S	Cu	Ni	Cr	Mo	V	Tl	Al	N	Nb	Zr	C_row_
0.134	1.460	0.330	0.018	0.007	0.330	0.040	0.500	0.005	0.006	0.002	0.046	0.006	0.003	0.002	0.504

**Table 2:** The welding wire AUTROD 13.36 chemical composition used for samples’ preparation.

Alloying Elements [%]												
C	Mn	Si	P	S	Cu	Ni	Cr	Mo	V	Al	N	Nb
0.116	1.487	0.536	0.015	0.011	0.383	0.631	0.233	0.028	0.004	0.012	0.014	0.002

**Table 3:** The welding electrode NICOR chemical composition used for samples’ preparation.

Alloying Elements [%]															
C	Mn	Si	P	S	Cu	Ni	Cr	Mo	V	Al	Zr	Nb	Ti	Sn	As
0.082	1.360	0.840	0.007	0.005	0.180	0.700	0.250	0.020	0.008	0.006	0.002	0.002	0.003	0.007	0.004

**Table 4:** Measurement of samples aged in a salt spray chamber (section loss) corrosion losses.

	Sample Thickness Survey				Sample Thickness Loss			
Survey No.					Survey No.			
	1	2	3	4	1	2	3	4
	[mm]	[mm]	[mm]	[mm]				
Preliminary	8.48	8.5	8.41	8.5				
0	8.16	8.17	8.16	8.18	3.77%	3.88%	2.97%	3.76%
I	8.03	8.09	8.01	8.18	5.31%	4.82%	4.76%	3.76%
II	8.08	8.16	8.17	8.11	4.72%	4.00%	2.85%	4.59%
Mean value	8.09	8.14	8.11	8.16	4.60%	4.24%	3.53%	4.04%
Sample count	3							
Standard deviation	0.054	0.036	0.073	0.033	0.63%	0.42%	0.87%	0.39%

**Table 5:** Measurement of (mass loss) corrosion losses of samples aged in a salt spray chamber.

	Before Aging			After Aging		
	Survey No.			Survey No.		
	1	2	3	1	2	3
	[g]	[g]	[g]	[g]	[g]	[g]
0	243.84	243.85	243.86	225.63	225.63	225.62
I	220.09	220.11	220.09	207.84	207.84	207.85
II	204.32	204.32	204.31	194.89	194.87	194.89
Mean value	222.75	222.76	222.75	209.45	209.45	209.45
Sample count	3					
Standard deviation	16.24	16.25	16.26	12.60	12.61	12.60

**Table 6:** Impact test results in the heat-affected zone (HAZ).

Steel Sheet as Delivered						Steel Sheet After Aging			
No.	Sample	Impact Strength [J/cm^2^]			No.	Sample	Impact Strength [J/cm^2^]		
		Single	Mean Value	Standard Deviation			Single	Mean Value	Standard Deviation
1	CV 1/2 KV1	35	47.3	8.7	13	CV 1/1 KV1	50	69.3	23.2
2	CV 1/2 KV2	54			14	CV 1/1 KV2	102		
3	CV 1/2 KV3	53			15	CV 1/1 KV3	56		
4	CV 1/4 KV1	48	47.3	9.0	16	CV 1/3 KV1	77	91.0	15.1
5	CV 1/4 KV2	58			17	CV 1/3 KV2	84		
6	CV 1/4 KV3	36			18	CV 1/3 KV3	112		
7	CV 100/2 KV1	164	144.0	24.8	19	CV 100/1 KV1	102	111.0	6.4
8	CV 100/2 KV2	109			20	CV 100/1 KV2	116		
9	CV 100/2 KV3	159			21	CV 100/1 KV3	115		
10	CV 100/4 KV1	129	96.3	30.3	22	CV 100/3 KV1	75	86.7	11.3
11	CV 100/4 KV2	56			23	CV 100/3 KV2	102		
12	CV 100/4 KV3	104			24	CV 100/3 KV3	83		

**Table 7:** Impact test results in the weld.

Steel Sheet as Delivered						Steel Sheet After Aging			
No.	Sample	Impact Strength [J/cm^2^]			No.	Sample	Impact Strength [J/cm^2^]		
		Single	Mean Value	Standard Deviation			Single	Mean Value	Standard Deviation
1	CV 1/2 KV1	91	85.3	4.2	25	CV 1/1 KV1	52	52.3	0.5
2	CV 1/2 KV2	81							
3	CV 1/2 KV3	84			26	CV 1/1 KV2	53		
4	CV 1/2 KV11	80	75.0	7.1					
5	CV 1/2 KV21	80			27	CV 1/1 KV3	52		
6	CV 1/2 KV31	65							
7	CV 1/4 KV1	68	78.0	7.3	28	CV 1/3 KV1	42	55.0	12.6
8	CV 1/4 KV2	81							
9	CV 1/4 KV3	85			29	CV 1/3 KV2	51		
10	CV 1/4 KV11	83	75.7	5.4					
11	CV 1/4 KV21	70			30	CV 1/3 KV3	72		
12	CV 1/4 KV31	74							
13	CV 100/2 KV1	70	64.3	4.5	31	CV 100/1 KV1	59	53.7	6.2
14	CV 100/2 KV2	59							
15	CV 100/2 KV3	64			32	CV 100/1 KV2	45		
16	CV 100/2 KV11	93	78.7	10.2					
17	CV 100/2 KV21	70			33	CV 100/1 KV3	57		
18	CV 100/2 KV31	73							
19	CV 100/4 KV1	138	122.0	14.4	34	CV 100/3 KV1	115	94.0	16.4
20	CV 100/4 KV2	103							
21	CV 100/4 KV3	125			35	CV 100/3 KV2	75		
22	CV 100/4 KV11	84	81.3	1.9					
23	CV 100/4 KV21	80			36	CV 100/3 KV3	92		
24	CV 100/4 KV31	80							

## Data Availability

The original contributions presented in this study are included in the article.
